# Unhealthy Levels of Phthalates and Bisphenol A in Mexican Pregnant Women with Gestational Diabetes and Its Association to Altered Expression of miRNAs Involved with Metabolic Disease

**DOI:** 10.3390/ijms20133343

**Published:** 2019-07-07

**Authors:** Alejandra Martínez-Ibarra, Luis Daniel Martínez-Razo, Edgar Ricardo Vázquez-Martínez, Nayeli Martínez-Cruz, Rogelio Flores-Ramírez, Elizabeth García-Gómez, Marisol López-López, Carlos Ortega-González, Ignacio Camacho-Arroyo, Marco Cerbón

**Affiliations:** 1 Doctorado en Ciencias Biológicas y de la Salud, Universidad Autónoma Metropolitana, Ciudad de México 04960, México; 2 Unidad de Investigación en Reproducción Humana, Instituto Nacional de Perinatología “Isidro Espinosa de los Reyes” – Facultad de Química, Universidad Nacional Autónoma de México, Ciudad de México 11000, México; 3 Coordinación del Servicio de Endocrinología, Instituto Nacional de Perinatología “Isidro Espinosa de los Reyes”, Ciudad de México 11000, México; 4 Coordinación para la Innovación y Aplicación de la Ciencia y la Tecnología, Universidad Autónoma de San Luis Potosí, San Luis Potosí 78210, México; 5 Departamento de Sistemas Biológicos, Universidad Autónoma Metropolitana, Ciudad de México 04960, México

**Keywords:** gestational diabetes, circulating miRNAs, phthalates, bisphenol A, endocrine disruptors, urinary metabolites

## Abstract

Several studies indicate that bisphenol A (BPA) and phthalates may have a role in the development of metabolic diseases using different molecular pathways, including epigenetic regulatory mechanisms. However, it is unclear whether exposure to these chemicals modifies serum levels of miRNAs associated with gestational diabetes mellitus (GDM) risk. In the present study, we evaluated the serum levels of miRNAs associated with GDM (miR-9-5p, miR-16-5p, miR-29a-3p and miR-330-3p) and urinary levels of phthalate metabolites (mono-n-butyl phthalate (MBP), mono-isobutyl phthalate (MiBP), mono-benzyl phthalate (MBzP) and mono(2-ethyl hexyl) phthalate (MEHP)) and bisphenol A in GDM patients and women without GDM during the second trimester of gestation. We observed higher levels of miR-9-5p, miR-29a-3p and miR-330-3p in sera of patients with GDM compared to non-diabetic subjects. Phthalates were detected in 97–100% of urine samples, while BPA only in 40%. Urinary MEHP and BPA concentrations were remarkably higher in both study groups compared to previously reported data. Unadjusted MEHP levels and adjusted BPA levels were higher in non-diabetics than in GDM patients (*p* = 0.03, *p* = 0.02). We found positive correlations between adjusted urinary MBzP levels and miR-16-5p expression levels (*p* < 0.05), adjusted MEHP concentrations and miR-29a-3p expression levels (*p* < 0.05). We also found negative correlations between unadjusted and adjusted MBP concentrations and miR-29a-3p expression levels (*p* < 0.0001, *p* < 0.05), unadjusted MiBP concentrations and miR-29a-3p expression levels (*p* < 0.01). Urinary MEHP levels reflect a striking exposure to di(2-ethylhexyl) phthalate (DEHP) in pregnant Mexican women. This study highlights the need for a regulatory strategy in the manufacture of several items containing endocrine disruptors in order to avoid involuntary ingestion of these compounds in the Mexican population.

## 1. Introduction

Gestational diabetes mellitus (GDM) is defined as a carbohydrate intolerance that results in hyperglycemia [1] and is first diagnosed during the second or third trimester of gestation in a woman who had neither pre-existing type 1 nor type 2 diabetes [2]. GDM occurs if pancreatic β-cells are unable to face the increased insulin demand during pregnancy [3]. Although GDM frequency varies among populations, it affects 10–25% of the worldwide population [4]. The prevalence of GDM has increased by ~10–100% in several ethnic groups during the last two decades [5]. In Mexico, the prevalence of GDM increased from 10% to 20% in the last decade alone [6,7]. Mexican women have an increased risk of developing GDM since Hispanic/Latino ethnicity is an important predisposing risk factor [8,9]. Furthermore, the prevalence of overweight-obesity in pregnant Mexican women is 63.19%, which increases the risk of developing GDM [10,11]. In addition, it is important to mention that women who are diagnosed with GDM are at an increased risk for developing type 2 diabetes mellitus (T2DM) within the five years of delivery [12,13].

There is growing evidence for the role of endocrine disrupting compounds (EDCs) in the development of metabolic diseases, especially when exposure occurs during critical periods of development such as in pregnancy or early life [14]. Bisphenol A (BPA) and phthalates are EDCs widely used in the manufacture of plastic food containers and bottles, food packaging, metal food cans, detergents, flame retardants, toys, cosmetics, pesticides, medical devices, among others [15].

Several studies carried out in non-pregnant populations and in animal models have demonstrated that exposure to some phthalates and BPA contribute to the development of obesity and glucose metabolism disorders [16,17,18]. Other studies have found correlations between high urinary levels of BPA and phthalates and adverse effects for both mothers and their offspring, including preterm delivery, metabolic dysfunction and altered newborn somatometric parameters [19,20,21,22,23].

Recently, a study reported a modified epigenetic regulation of microRNAs (miRNAs) induced by BPA and phthalates [24]. miRNAs are single-stranded, short non-coding RNA sequences (~22 nucleotides) that regulate gene expression at the posttranscriptional level through base-pairing with complementary sequences of the 3′untranslated region (UTR) of messenger RNAs (mRNA), leading to translational repression or mRNA degradation [25]. Several miRNAs, also known as circulating miRNAs or extracellular miRNAs, have been detected in different biological fluids, including plasma and serum [26].

Several studies have identified multiple dysregulated circulating miRNAs in patients diagnosed with GDM during pregnancy [27,28]. Particularly, Zhao and cols. found that miR-29a-3p was significantly decreased through gestational weeks 16–19 and reported that *Insig1* (Insulin-induced gene 1) is a target of miR-29a-39, validated in HepG2 cells in Insig1 protein expression assays [27]. Another study evaluated miRNA expression in plasma collected between the 16th and 19th gestational week in Chinese women, and demonstrated that miR-16-5p was significantly up-regulated in diabetic pregnant women with respect to controls. This miRNA was mainly associated with MAPK, insulin, TGF-β, and mTOR signaling pathways [28].

In another study, Li et al. identified miR-9-5p in full-term-placentas as a miRNA associated with GDM and an increased risk of macrosomia [29]. Up-regulation of miR-330-3p was found in plasma of GDM patients at gestational weeks 24–33 when compared to non-diabetic women. Moreover, miR-330-3p predicted target genes were related to proliferation and differentiation of beta-cells, and insulin secretion [30].

Although there is vast evidence suggesting that exposure to BPA and phthalates is associated with metabolic disorders that occur in gestation, it is unclear whether exposure to these chemicals modifies serum levels of miRNAs associated with GDM. These changes may be related to a significant increase in the prevalence of the disease. Particularly in Mexico, there is a high prevalence of GDM, as well as a lack of regulation in the use of toxic substances such as phthalates and BPA. Importantly, few studies have focused on the relationship between the exposure of these compounds and the development of GDM.

The aim of this study was to evaluate the expression of four miRNAs related to GDM development in pregnant Mexican women, and whether these are associated with total BPA and MBP, MiBP, MBzP and MEHP phthalate metabolites levels in urine samples.

## 2. Materials and Methods

### 2.1. Participants and Samples

Patients were recruited at the Instituto Nacional de Perinatología “Isidro Espinosa de los Reyes” (INPer) in Mexico City. Women with singleton gestations in the second trimester of pregnancy were invited to participate in the study during prenatal visits to endocrinology medical service. After signing an informed consent, 40 pregnant Mexican women with ages 24–45 years in the second third of gestation were included in this study; 18 women with GDM and 22 non-diabetics (control group).

Inclusion criteria for the GDM group were pregnant women with GDM diagnosis in accordance to the International Association of Diabetes Pregnancy Study Group (IADPSG) criteria, or women without impaired glucose tolerance (non-diabetic group). All pregnant women were screened for GDM by a 75-g two-hour oral glucose tolerance test (OGTT). Exclusion criteria for both groups were multiple pregnancies, women with autoimmune, hematologic and chronic systemic diseases, arterial hypertension, treated neurological disorders, polycystic ovary syndrome, hypothyroidism, as well as women with smoking or drinking habits.

Blood and urine samples were obtained from all patients. Serum was separated from whole blood samples by centrifugation at 3000 rpm (1900 g) for 10 min at room temperature. Serum samples were collected in RNase-free tubes and centrifuged twice at 12,300 rpm (16,000 g) for 10 min at 4 °C. Finally, serum samples were aliquoted and stored at −80 °C until RNA extraction. Single urine samples from each patient were collected in amber glass containers with polypropylene cap and stored at −20 °C for determination of BPA and phthalate metabolites and creatinine. Urinary BPA and phthalate metabolite measurements were carried out in the Laboratorio de Salud Total from the Coordinación para la Innovación y Aplicación de la Ciencia y la Tecnología, at the Universidad Autónoma de San Luis Potosí, (CIACyT – USLP), in San Luis Potosí, Mexico. This study was approved by the Committee of Ethics in Research and Biosecurity of the Instituto Nacional de Perinatología “Isidro Espinosa de los Reyes” ((548) 212250-3000-21402-02-16; 30-September-2016).

### 2.2. RNA Extraction

Total RNA was extracted from serum using a commercial column-based system (Qiagen MiRNeasy Serum/Plasma Kit, Hilden, Germany) following the manufacturer’s instructions with the following modifications. Serum samples were thawed on ice and then further centrifuged 12,300 rpm (16,000 g) for 10 min at 4 °C in order to completely remove cell debris. An aliquot of 400 μL of serum per sample was transferred to a new microcentrifuge tube and 1200 μL of Trizol LS Reagent (Invitrogen; Thermo Fisher Scientific, Inc., Waltham, MA, USA) was added. Subsequent protocol steps were followed according to manufacturer’s instructions. Finally, RNA was eluted in 20 µL of RNase-free water. The quality and yield of isolated RNA were determined by Qubit (Thermo Scientific, Waltham, MA, USA).

### 2.3. miRNA Expression

Total RNA was reverse transcribed into cDNAs with TaqMan Advanced miRNA cDNA Synthesis kit (Thermo Scientific, Waltham, MA, USA). miRNA expression assay was performed with the Applied Biosystems StepOnePlus Real-Time PCR System (Life Technologies, Grand Island, NY, USA) using TaqMan Fast Advanced Master Mix (Thermo Scientific, Waltham, MA, USA) and stem-loops probes of TaqMan Advanced MicroRNA Assays (Thermo Scientific, Waltham, MA, USA) following manufacturer’s instructions. We selected four miRNAs previously related to GDM development: miR-9-5p, miR-16-5p, miR-29a-3p and miR-330-3p (Assay IDs 478214, 477860, 478587 and 478030). Selected miRNAs were analyzed in the serum of 22 GDM patients and 18 non-diabetic women. All miRNAs were measured by triplicate and negative controls without cDNA and with non-retrotranscribed RNA were included in all experiments. Normalization was performed with miR-454 serving as an endogenous control [31], and relative expression of target miRNAs was determined by the ΔΔCT method.

### 2.4. Phthalates Metabolites and Total BPA Assessment

Forty maternal urine samples were analyzed for MEHP, MBzP, MBP, MiBP, and total BPA at the Laboratorio de Salud Total from the Coordinación para la Innovación y Aplicación de la Ciencia y la Tecnología at the Universidad Autónoma de San Luis Potosí (CIACyT – USLP), in San Luis Potosí, Mexico. Urine samples were processed by enzymatic deconjugation of the glucuronides of phthalate monoesters and BPA followed by solid phase extraction. Identification and quantification of MEHP, MBzP, MBP, MiBP and total BPA were performed by reverse phase Ultra-performance liquid chromatography-tandem mass spectrometry (UPLC-MS/MS), in accordance to CDC’s Laboratory Procedure Manuals for quantification of phthalates and BPA in urine [32,33].

Limits of detection (LOD) for metabolites were 6.41, 0.23, 1.08, 0.15 and 22.2 ng/mL for BPA, MiBP, MBP, MBzP, and MEHP respectively. For concentrations below the LOD, a value equal to each sample’s specific LOD divided by the square root of 2 was used. All urinary phthalate and BPA metabolite levels were adjusted for dilution using urinary creatinine values. More details about chromatographic conditions, sample concentrations (Excel file), and chromatograms (PDF file; pages 1–56 for BPA, pages 57–182 for phthalate metabolites) are provided in the Supplementary Materials.

**Figure 1:**
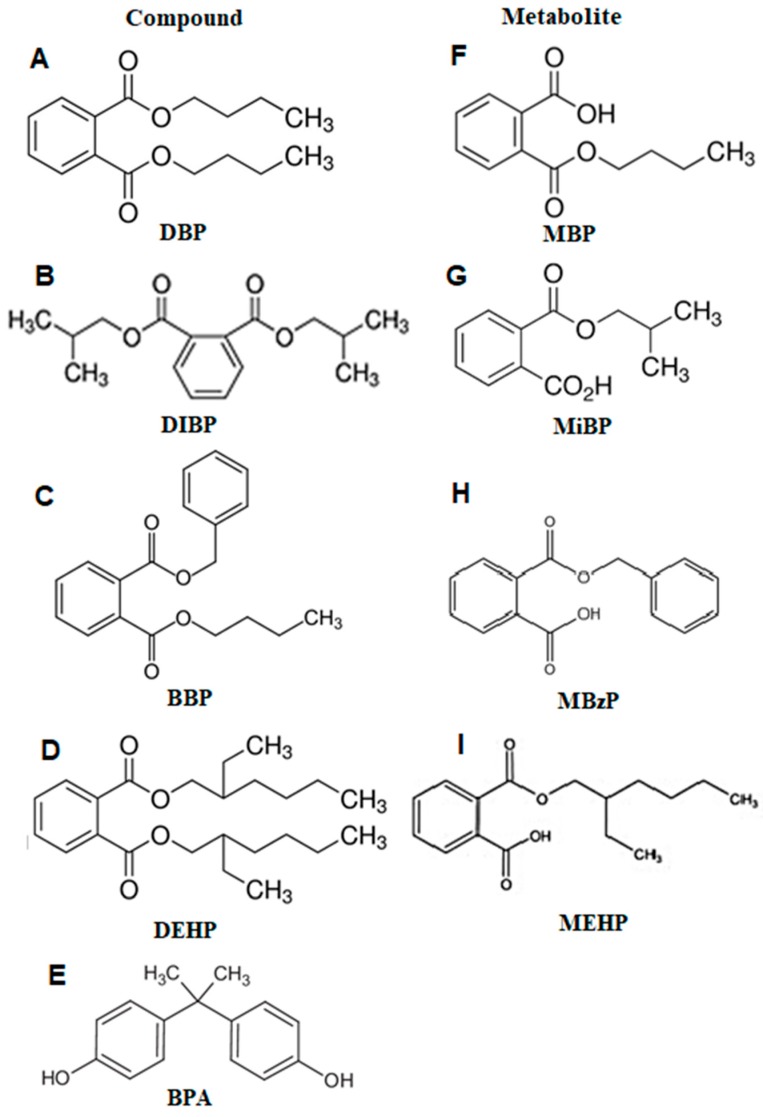
Chemical structures of phthalates (**A**–**D**), their metabolites (**F**–**I**) and BPA (**E**). DBP, Dibutyl phthalate. DIBP, Diisobutyl phthalate. BBP, Benzyl butyl phthalate. DEHP, di(2-ethylhexyl) phthalate. BPA, Bisphenol A. MBP, Mono-n-butyl phthalate. MiBP, Mono-isobutyl phthalate. MBzP, Mono-benzyl phthalate. MEHP, Mono(2-ethyl hexyl) phthalate.

The chemical structures of the phthalate metabolites analyzed, their respective precursor compounds and BPA are depicted in Figure 1.

### 2.5. Data Analysis

Student’s *t*-test and *X^2^* test were used to compare population characteristics between both study groups. Analysis of variance (ANOVA) was used for multiple comparisons. Analyses of differences in urinary levels of phthalates and BPA levels in urine were conducted using Mann-Whitney *U*-test. Spearman correlation was used to explore the association between miRNA expression levels and biochemical parameters as well as to evaluate the association between phthalate and BPA urinary concentrations, miRNA expression levels and biochemical parameters. Data analysis were performed using GraphPad Prism version 5.0 for Windows (GraphPad Software, La Jolla, CA, USA). Values of *p* < 0.05 were considered to be statistically significant.

## 3. Results

### 3.1. Population Characteristics

Population clinical data are summarized in Table 1. No statistical differences were found in maternal age, pregestational body mass index and pregnancy length between non-diabetic women and GDM patients. Women with GDM had higher parity compared to non-diabetic women. As expected, average glucose values at fasting (*p* ≤ 0.0001), one-hour (*p* ≤ 0.0001) and two-hour (*p* ≤ 0.0001) OGTT were significantly higher in GDM women compared to non-diabetics.

### 3.2. Up-Regulation of miR-9-5p, miR-29a-3p, and miR-330-3p in GDM Serum Samples of GDM Women

**Figure 2:**
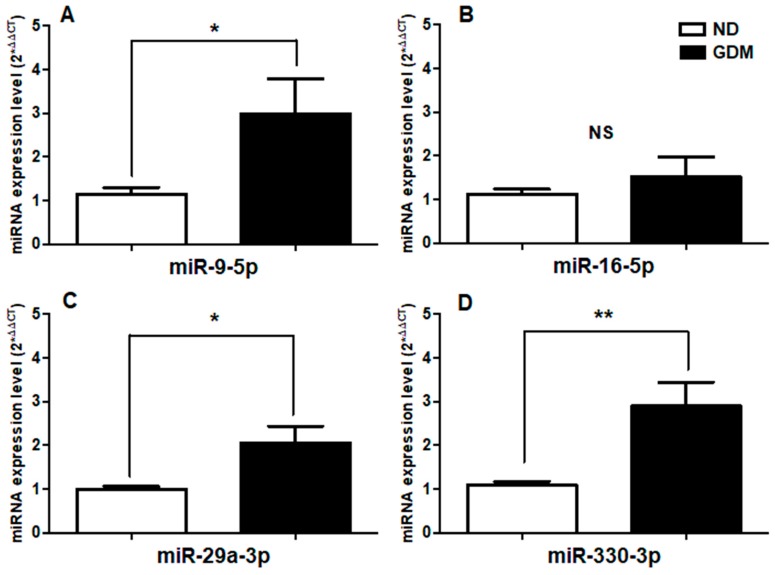
Changes in the relative expression of circulating miRNAs in pregnant women with GDM. mir-454 was used as endogenous control. ND, Non-diabetic patients. GDM, Patients with gestational diabetes mellitus. (**A**) miR-9-5p, (**B**) miR-16-5p. (**C**) miR-29a-3p. (**D**) miR-330-3p. The data shown correspond to the mean values + standard error mean. The statistical significance was determined by Mann-Whitney *U*-Test. * *p* < 0.05, ** *p* < 0.01, NS. not significant.

Relative expression levels of miR-9-5p, miR-29a-3p and miR-330-3p were significantly up-regulated in serum from GDM patients (*p* = 0.03, 0.01, and 0.004, respectively) compared to serum from non-diabetic women (Figure 2A,C,D) while no significant differences were observed in miR-16-5p expression levels (Figure 2B).

### 3.3. Phthalates Metabolites and Total BPA Urine Levels in Second Trimester Pregnant Women

Limits of detection (LOD), geometric means, 95% confidence intervals (95% CI), percentiles, and minimum and maximum adjusted and unadjusted concentrations of all analyzed samples are described in Table 2. The detectable rate of the four analyzed phthalate metabolites and total BPA in all urine samples are as follows: MBzP, MBP, and MEHP (100%); MiBP (97%) and BPA (35%). The detection rate of the analyzed metabolites in urine samples of both non-diabetic women and women with GDM was similar (Table 3). MEHP was the most abundant metabolite found in all urine samples, followed by MBP, BPA, MiBP, and MBzP (Table 2). Statistically significant differences were not found in MBzP, MBP, MiBP and BPA unadjusted levels between groups, except MEHP levels that were higher in non-diabetics compared to women with GDM (*p* = 0.03) (Table 3). However, urinary creatinine-adjusted concentrations of all metabolites, except BPA, were not significantly different among groups. Lower levels of BPA were detected in urine samples from women with GDM compared to non-diabetic women (*p* = 0.02) (Table 3).

### 3.4. Correlation between Phthalate Metabolites, Total BPA Urine Levels and Serum miRNA Expression Levels, and Clinical Variables

The association analyses of phthalates and BPA urinary levels showed positive correlations between adjusted urinary MBzP levels and miR-16-5p expression levels (*p* < 0.05), and adjusted MEHP concentrations and miR-29a-3p expression levels (*p* < 0.05). We also found negative correlations between unadjusted and adjusted MBP concentrations and miR-29a-3p expression levels (*p* < 0.0001, *p* < 0.05), unadjusted MiBP concentrations and miR-29a-3p expression levels (*p* < 0.01). Moreover, BPA unadjusted urinary concentrations had a positive relationship with pregestational body mass index (BMI) (*p* < 0.01). Data is shown in Table 4.

## 4. Discussion

Exposure to EDCs such as BPA and phthalates has been previously associated with several metabolic disorders. Studies have been performed extensively in non-pregnant populations, but less studied in pregnant women. GDM is one of the most common metabolic disorders that occurs during pregnancy in our population, with detrimental effects for both mothers and newborns. Studies exploring associations between BPA and phthalates exposure and GDM are scarce. However, recent evidence suggests that microRNAs are an underlying mechanism that may provide a better understanding of the molecular contribution of EDCs in the physiopathology of GDM. miRNAs control metabolic processes in various tissues by regulating the expression of genes that code for proteins responsible for metabolism. Dysregulation of microRNA expression has been associated with obesity, T2DM and GDM [34,35].

In the present study, we analyzed total urinary BPA and MBP, MiBP, MBzP and MEHP metabolite levels, and miRNA expression linked to GDM during the second trimester of pregnancy. Our results found high levels of DEs in the urine of both non-diabetic pregnant women and women with GDM. Interestingly, we found correlations between EDC content and miRNA expression levels in non-diabetic patients in contrast to GDM patients.

To the best of our knowledge, this is the first study in Mexico to evaluate the expression of circulating miRNAs related to GDM. We found that GDM is associated with an increase in the expression of serum miR-9-5p, miR-29a-3p and miR-330-3p when compared to non-diabetic women during the second trimester of pregnancy. Interestingly, miRNA-9-5p has previously been found to be down-regulated in placental villous tissues and primary villous cytotrophoblasts (CTs) from women with GDM associated with risk of macrosomia [29]. HK-2 (Hexokinase-2) was also shown to be a direct target of miR-9-5p, which in turn contributes to GDM progression by regulating gene expression related to glycolytic pathways and the mitochondrial complex, as well leading to a decrease in the expression of GLUT1 (glucose transporter 1), PFK (phosphofructokinase) and LDH (lactate dehydrogenase), at both mRNA and protein levels [36]. Moreover, overexpression of miR-9-5p in serum was found in newly diagnosed T2DM patients compared to individuals with normal glucose tolerance [37]. This suggests that miR-9-5p may play an important role in the regulation of glucose metabolism. Up-regulation of miR-9-5p in serum of our GDM patients may reflect altered expression of this miRNA in placental tissue, and in turn, evidence the pathological condition of GDM. However, more studies should be performed to evaluate miR-9-5p expression in serum samples from woman with GDM.

We also found overexpression of miR-29-3p in serum from women with GDM during the second trimester of pregnancy. This miRNA was recently identified as a specific miRNA associated with GDM development in early gestation, but found down-regulated in serum collected at 16–19 gestational weeks from women with GDM compared to controls [27,38]. By contrast, our results are similar to those reported in a study carried out in a Chinese cohort where miR-29a levels were overexpressed in serum samples from newly diagnosed T2DM patients compared to T2DM-susceptible individuals with normal glucose tolerance [37]. Overexpression of miR-29a-3p has been related to altered glucose intake and insulin-stimulated glucose metabolism in skeletal muscle from T2DM patients. It modifies the expression of various genes involved in the insulin signaling pathway, such as HK2, and negatively regulates fatty acid oxidation through PGC1α expression [39,40]. Our findings support the idea that miR-29a-3p is an important molecule in the regulation of glucose metabolism and may be involved in insulin resistance in GDM patients.

Furthermore, we found that miR-330-3p levels were higher in serum from women with GDM compared with non-diabetics in the second trimester of pregnancy. Our results are consistent with a previous study, which also reported an association between an increase of miR-330-3p in plasma from GDM patients from the 24th–33rd week of pregnancy, a decrease in the insulinemia levels and a high rate of primary cesarean sections. Furthermore, this study predicted and validated target genes of miR-330-3p, which are related to proliferation and differentiation of beta cells and insulin secretion [30]. Therefore, our findings support involvement of miR-330-3p in GDM physiopathology due to its participation in glucose homeostasis.

Our results showed no significant differences in the expression of miR-16-5p between non-diabetic patients and GDM patients although it has previously been reported as a specific plasma miRNA related to GDM [28].

In our study, we observed differential expression of miRNAs related to GDM. These differences in miRNA expression in women with GDM have been previously associated with many factors. These include diverse patterns of expression found in different biological tissues and fluids [41], temporary regulation of miRNAs during gestational stages [42], racial/ethnic differences [43], and regulatory changes due to environmental factors, including exposure to various endocrine disrupting substances [25].

With regard to total BPA measurements, we detected a frequency of 40%, lower than the reported nearly 70% by the ELEMENT (Early Life Exposure in Mexico to Environmental Toxicants) study [19,44,45]. This study included women with GDM, preeclampsia and hypertensive disorders related to gestation during the first trimester of gestation or at delivery in Mexico City between 1997 and 2003 [19,44,45]. The average urinary BPA levels obtained in our study are shown in Table 5 along with values reported in previous studies in Mexico and other countries, including Canada, China, Korea and the United States [22,46,47,48,49,50,51].

**Figure 3:**
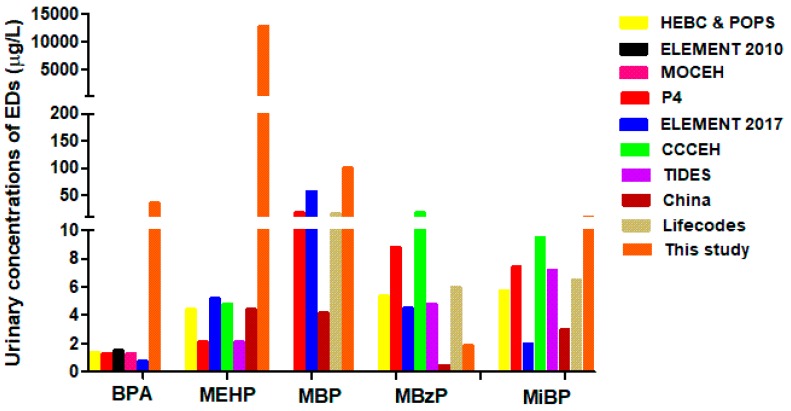
Comparison of median concentrations of total BPA and phthalates metabolites in urine from several cohorts.

Although the BPA detection frequency that we report in this study was lower compared to other studies, the average BPA concentration that we found was almost 30-fold higher than values reported by another study in Mexico City and several other studies in pregnant women around the world (Table 5, Figure 3). It is worrisome that the population in our study showed high levels of exposure to this compound. This is probably due to the consumption habits of products that contain BPA.

With respect to measurements of phthalate metabolites, we found MBP, MBzP and MEHP in samples of all pregnant women and MiBP was found in 98% of the studied population. This data is similar to that found in an earlier report where urinary levels of phthalate metabolites were found in 89–100% of Mexican pregnant women [19,45,46]. Urinary levels of MiBP and MBzP were similar to previous reports in Mexican pregnant women and in pregnant women of other countries (Table 5). However, urinary levels of MEHP and MBP were higher than in other reports from Mexico and other countries worldwide (Table 5). Urinary levels of MBP found in this study are two-fold higher than previous values reported in Mexican pregnant women [44,45].

Compared to other countries, urinary levels of MBP in this study are ten-fold higher than those found in recent reports in pregnant Canadian and American women and 20-fold higher than in pregnant Chinese women (Table 5, Figure 3). Remarkably, urinary levels of MEHP detected in this study are extremely higher compared to those found in earlier reports in pregnant women from Mexico City and in other populations worldwide (Table 5, Figure 3). In our study, MEHP urinary concentrations are 2600-fold higher than levels previously reported in Mexican pregnant women, as well as 2900-fold higher than levels reported in pregnant women from China, and almost 5000-fold higher than levels reported in pregnant women from Canada and USA, respectively (Figure 3). These findings suggest that Mexican pregnant women are overexposed to DBP (Figure 1A) and DEHP (Figure 1D) when compared to other populations. This may be due to lack of regulation in phthalate usage in Mexico in contrast to other countries like Canada, The United States of America, Australia, The European Union and some Asian countries where phthalate usage has been banned or reduced in some products [52,53].

Few studies have explored the relationship between exposure to BPA and phthalates and the occurrence of metabolic disorders during gestation, such as GDM [48,49,54]. In the present study, we found high unadjusted MEHP levels and high creatinine-adjusted BPA levels in non-diabetics compared to women with GDM (Table 3). Similar results were found in the Shanghai Obesity and Allergy Cohort study [55], where maternal urinary BPA was associated with a decreased risk of GDM and plasma glucose levels. This controversial result may be explained by the similarity in molecular structures of BPA and 17β-estradiol (E2). BPA can bind to estrogen receptors and produce effects similar to those of E2 [55], which at low concentrations (10–8 M) has shown to improve glucose tolerance through ERα/ERβ and ER coupled G protein (GPER) in murine models of T2DM [56]. An in vitro study reported that BPA concentrations of 100 µM increased insulin release from pancreatic β-cells INS-1E. Nevertheless, this concentration is almost 50-fold higher than BPA levels detected in urine samples from both groups in our study [57]. However, a growing number of epidemiological studies in non-pregnant populations and experimental studies have shown associations between BPA exposure and insulin resistance and glucose intolerance [58,59,60,61]. Thus, more association studies between BPA exposure and glucose metabolism during pregnancy are needed, considering levels of exposure to BPA in each population.

Our findings regarding high urinary levels of MEHP in non-diabetic women compared to GDM patients are similar to the results found in the Cambridge Baby Growth Study subcohort, where no positive associations between MEHP serum levels and GDM were found. However, positive associations between MEHP serum levels with 120-min plasma glucose in women without GDM [62] were found. In another study performed in a cohort of pregnant women in China, MEHP exposure levels were correlated with fasting blood glucose levels in the third trimester of pregnancy in non-diabetics but not in women with GDM [36]. An in vitro study reported that concentrations of 100 µM of MEHP increased insulin release from pancreatic β-cells INS-1E [58], which may indicate that this metabolite could be affecting glucose metabolism. Yet, MEHP concentration was two-fold higher than the urinary MEHP levels detected in the non-diabetics in our study. Nevertheless, we did not find an association between MEHP and GDM, nor has it been previously reported. Therefore, more studies should be conducted to explore MEHP effects on pregnant women.

To the best of our knowledge, we report for first time associations between urinary levels of some phthalates and expression levels of circulating miRNAs linked to GDM. We found a significant positive correlation between adjusted urinary levels of MBzP and expression levels of miR-16-5p in non-diabetic women (*r* = 0.4737, *p* < 0.05). In our study, we found no statistically significant differences in miR-16-5p expression levels and MBzP urinary concentrations among groups. The positive correlation found between the two may indicate that miR-16-5p could be used as a possible biomarker of exposure to MBzP. Metabolic alterations may be a confusing variable since no correlation was observed in the group of GDM patients.

We also found significant negative correlations between unadjusted and adjusted urinary levels of MBP and miR-29 levels expression (*r* = −0.7140, *p* < 0.0001; *r* = −0.5418, *p* < 0.05), and MiBP unadjusted levels and miR-29-3p levels expression (*r* = −0.6719, *p* < 0.01). These inverse correlations found in non-diabetic women would seem to indicate that high values of MBP and MiBP correspond to low values of miR-29a-3p expression. We found similar values of both MBP and MiBP in women with and without GDM. This seems to be consistent with the down-regulation of miR-29a-3p that we found in non-diabetic women.

MEHP adjusted levels correlated positively with miR-29a-3p expression levels (*r* = 0.4912, *p* < 0.05) in non-diabetic women, which would indicate that urinary concentrations of MEHP and values of miR-29a-3p expression in women without GDM correlate in a direct sense. Although our results might suggest that mir-29a is associated with GDM, this disease may be a confusing variable since no correlation was observed between this phthalate and miR-29a-3p in the group of GDM patients.

Furthermore, we found a significant positive correlation between unadjusted urinary levels of BPA (*r* = 0.8810, *p* < 0.01) and pregestational-BMI in women without GDM but not in GDM group. However, we found no differences in pregestational BMI among both groups. This relationship was probably not observed in the group of GDM patients due to the presence of the disease. Studies performed in rodents have reported that perinatal exposure to BPA is associated with an increased body weight and fat deposition in offspring [63,64,65]. In humans, prenatal exposure to high levels of BPA during pregnancy has been associated with decreased BMI, body fat, and overweight/obesity in female offspring [66]. We found higher BPA values in women without GDM compared to those found in women with GDM, although the proportion of positive samples to BPA in the non-diabetics was as low as 36%. We consider this a limiting factor in our study and further studies with a higher sample size should be performed.

Our results showed interesting associations between urinary levels of some phthalates and expression levels of circulating miRNAs linked to GDM. However, it is important to point out that both phthalates and BPA are capable of interacting with hormone receptor proteins or with enzymes involved in the synthesis or activation of hormones. Therefore, they can also produce their effects through several mechanisms, such as activation of peroxisome proliferator-activated receptors (PPARα,γ), and nuclear receptors that play a key role in the regulation of adipocyte differentiation and adipogenesis, lipid metabolism and glucose homeostasis by the modulation of insulin sensitivity [16,55]. Thus, various mechanisms could be involved in the pathogenesis of GDM.

Aspects that may influence the consistency in findings of levels of environmental exposure to EDCs include population size, timing of sample collection, changes in xenobiotic metabolism during various stages of pregnancy [67], ethnicity/racial differences [68], lifestyle, consumer habits and different sources of exposure among others.

## 5. Conclusions

In the present study, a significant up-regulation of miR-9-5p, miR-29a-3p y miR-330-3p was found in women with GDM. Whether these microRNAs can be used as GDM biomarkers in the Mexican population remains to be further explored by increasing the representative sample size.

MEHP was the most abundant metabolite found in all urine samples, followed by MBP, BPA, MiBP, and MBzP. MEHP unadjusted levels and BPA adjusted levels were higher in non-diabetics. MEHP urinary concentrations were 2600-fold higher than previously reported levels in pregnant Mexican women and almost 5000-fold higher than levels reported in other populations of pregnant women. BPA concentrations were almost 30-fold higher than values reported by a previous study in Mexico City and several studies in pregnant women around the world.

We report for first-time correlations between urinary levels of some phthalates and miRNA expression levels linked to GDM: adjusted MBzP urinary concentrations correlated with miR-16-5p expression levels; expression levels of miR-29a-3p correlated with non-adjusted and adjusted urinary MBP concentrations, adjusted urinary MEHP concentrations and unadjusted urinary MiBP concentrations. Unadjusted urinary BPA concentrations correlated with pre-gestational body mass index. Further studies are needed to confirm these findings and to explore associations between gestational exposure to phthalates and BPA, and the risk of developing GDM.

This study highlights the need for a regulatory strategy in the manufacture of several items containing endocrine disruptors in order to avoid the involuntary ingestion of these compounds by the Mexican population.

## Figures and Tables

**Table 1:** Population characteristics.

Parameter	Nondiabetic Pregnant Women (*n* = 22)	Pregnant Women with GDM (*n* = 18)	*p* Value
Maternal Age (years)	31.86 ± 5.1	34.11 ± 4.5	0.15
Weeks of gestation at sampling	18.92 ± 6.13	23.25 ± 5.43	0.13
Pregestational BMI (kg/m^2^)	30.15 ± 6. 6	29.47 ± 5.2	0.72
Parity	2.0 ± 1.3	2.6 ± 0.9	0.02
First	45%	11.1%	
Second	35%	33.3%	
Third or more	20%	55.6%	
Pregnancy length (weeks)	38.56 ± 1.57	37.39 ± 2.55	0.13
OGTC (75 g)			
Fasting plasma glucose (mg/dL)	77.76 ± 9.3	99.89 ± 2.91	<0.0001
1 h plasma glucose (mg/dL)	129.6 ± 28.44	207.3 ± 24.87	<0.0001
2 h plasma glucose (mg/dL)	176.6 ± 17.93	180.0 ± 27.65	<0.0001

Data are expressed as mean ± standard deviation (SD) or percentage.

**Table 2:** Urinary concentrations of unadjusted (µg/L) and adjusted total BPA and phthalates metabolites (µg/g Cr) for all urine samples.

Chemical	GM	Minimum	Percentiles				Maximum
	(95% CI)		25th	50th	75th	90th	
*Unadjusted*							
BPA	35.22 (21.80–56.91)	4.9	22.9	39.10	67.70	112.4	123.9
MBzP	1.86 (1.26–2.73)	0.5	0.5	1.75	4.82	11.88	46.5
MBP	101.3 (78.48–130.8)	24.0	58.73	105.3	153.4	243.2	1012
MiBP	10.35 (7.35–14.56)	0.6	5.0	10.50	18.30	41.6	99.60
MEHP	12,837 (11426–14422)	4976	10,648	13,049	15,814	20,675	24,524
*Adjusted*							
BPA	31.19 (20.71–46.99)	8.2	20.09	39.28	49.69	77.15	77.92
MBzP	1.71 (1.25–2.33)	0.33	0.73	1.7	3.44	8.77	12.63
MBP	93.15 (81.14–106.9)	35.43	64.20	97.34	118.2	174.5	210.4
MiBP	9.89 (7.68–12.75)	1.15	6.29	10.36	16.62	29.49	46.03
MEHP	11,732 (9341–14,735)	2141	7681	10,711	18,388	32,689	47,071

N = 40; GM, Geometric mean; LOD, Limit of detection.

**Table 3:** Urinary concentrations of unadjusted (µg/L) and adjusted total BPA and phthalates metabolites (µg/g Cr) by group.

Chemical	ND (*n* = 22)	GDM (*n* = 18)	ND	GDM	*p* value
	Detection Frequency (%)	Detection Frequency (%)	GM (95% CI)	GM (95% CI)	
*Unadjusted*					
BPA	36.3	33	47.86 (26.94–85.03)	23.4 (9.24–59.26)	0.18
MBzP	100	100	1.83 (1.09–3.06)	1.58 (0.92–2.7)	0.67
MBP	100	100	102.7 (72.74–145.0)	87.75 (62.79–122.6)	0.53
MiBP	100	94.4	9.09 (5.41–15.27)	9.7 (6.52–14.43)	0.81
MEHP	100	100	13,135 (10,861–15,884)	11,586 (10,408–12,898)	0.03
*Adjusted*					
BPA	36.3	33	46.08 (30.99–68.52)	19.79 (10.01–39.09)	0.02
MBzP	100	100	1.75 (1.1–2.78)	1.66 (1.05–2.61)	0.98
MBP	100	100	94.22 (77.4–114.7)	91.97 (74.22–114.0)	0.94
MiBP	100	94.4	8.95 (6.06–13.23)	11.19 (7.99–15.65)	0.42
MEHP	100	100	11,855 (8200–17,138)	11,582 (8778–15,282)	0.72

ND, Non-diabetic patients. GDM, Patients with gestational diabetes mellitus. 95% CI, 95% confidence intervals.

**Table 4:** Spearman correlation coefficients between unadjusted (µg/L) and adjusted (µg/g Cr) urinary concentrations of total BPA and phthalates and relative expression of circulating miRNAs and Pregestational BMI.

Chemical	mir-16		mir-29a		Pregestational BMI	
	Unadjusted	Adjusted	Unadjusted	Adjusted	Unadjusted	Adjusted
**MBzP**						
ND	0.2211	0.4737 *	−0.2496	0.01053	−0.03088	−0.04545
GDM	0.3274	0.1961	0.2107	0.1084	0.2233	0.1373
**MBP**						
ND	0.07544	0.1744	−0.7140 ***	−0.5418 *	−0.1545	−0.2692
GDM	0.06127	−0.2328	0.1785	−0.1517	0.2425	0.01342
**MiBP**						
ND	−0.2737	−0.1596	−0.6719 **	−0.3789	0.1403	0.1870
GDM	−0.04290	−0.06176	0.4165	0.1005	−0.1104	−0.3382
**MEHP**						
ND	−0.3947	−0.1035	0.2947	0.4912 *	−0.08182	−0.09351
GDM	−0.05882	−0.3627	−0.05882	−0.2735	0.7143	−0.1723
**BPA**						
ND	0.1786	0.7143	0.08571	0.5000	0.8810 **	0.5714
GDM	0.02857	−0.02857	−0.4857	−0.6571	0.7143	0.4857

ND, Non-diabetic patients. GDM, Patients with gestational diabetes mellitus. GM, Geometric mean. * *p* < 0.05, ** *p* < 0.01, *** *p* < 0.0001.

**Table 5:** Comparison of the median urinary concentrations of total BPA and phthalate metabolites in studies of pregnant women.

Population	Country	Trimester	GM (μg/L)					Ref.
			BPA	MEHP	MBP	MBzP	MiBP	
This study (*N = 40*)	Mexico	2nd	35.22	12837	101.3	1.86	10.35	
**Harvard Epigenetic Birth Cohort (HEBC) and the Predictors of Preeclampsia Study (POPS) *(N = 179)***	USA	1st	1.36	4.38	-	5.34	5.74	[46]
**The Plastics and Personal-care Products use in Pregnancy (P4) study *(N = 70)***	Canada	1st	1.14	2.24	16.2	9.85	7.40	[47]
		2nd	1.31	2.09	19.16	8.80	-	
		3rd	1.31	2.26	22.63	10.70	-	
**Early Life Exposure in Mexico to Environmental Toxicants (ELEMENT study) *(N = 60)***	Mexico	3rd	1.52	-	-	-	-	[19]
**Mothers and Children’s Environmental Health (MOCEH) study *(N = 757)***	Korea	3rd	1.29	-	-	-	-	[22]
**The Early Life in Mexico to Environmental Toxicants (ELEMENT Study) *(N = 249)***	Mexico	3rd	0.74	5.20	56.90	4.50	1.99	[44]
**Columbia Center for Children’s Environmental Health (CCCEH) cohort *(N = 246)***	USA	3rd	-	4.80	-	17.50	9.50	[48]
**The Infant Development and Environment Study (TIDES) *(N = 668)***	USA	1st	-	2.50	-	4.30	5.20	[49]
		3rd	-	2.10	-	4.80	7.20	
**Unnamed Cohort *(N = 30)***	China	3rd	-	4.43	4.15	0.46	2.96	[50]
**LifeCodes Study *(N = 350)***	USA	1st	-	-	14.20	5.50	6.10	[51]
		2nd	-	-	15.20	6.00	6.50	
		3rd	-	-	16.30	6.70	7.00	

GM. Geometric mean.

## Supplementary Materials

Supplementary materials can be found at https://www.mdpi.com/1422-0067/20/13/3343/s1. Supplementary material for this article includes chromatograms, analytical conditions and validation of the method used for the phthalates metabolites and total BPA assessment.
